# Frontal Sinus Osteoma Causing Tension Pneumocephalus and Acute Hemiparesis: A Rare Neurological Complication

**DOI:** 10.7759/cureus.87707

**Published:** 2025-07-11

**Authors:** Vikrant Keshri, Jotdeep S Bamrah, Aiman Maidan

**Affiliations:** 1 Neurosurgery, Smt. B. K. Shah (SBKS) Medical Institute and Research Centre, Sumandeep Vidyapeeth Deemed to be University, Vadodara, IND; 2 Neurosurgery, Taraz City Multidisciplinary Hospital and Consulting and Diagnostic Center, Taraz, KAZ

**Keywords:** frontal sinus osteoma, hemiparesis, neurosurgical emergency, paranasal sinus tumor, tension pneumocephalus

## Abstract

Frontal sinus osteomas are typically benign and asymptomatic lesions. However, in rare instances, they can erode the posterior sinus wall, leading to tension pneumocephalus, a neurosurgical emergency. We report the case of a 33-year-old female presenting with progressive headache and right-sided hemiparesis. MRI and CT imaging revealed a large frontal sinus osteoma eroding the posterior table and resulting in tension pneumocephalus. The patient underwent frontal craniotomy, osteoma excision, dural repair, and sinus exteriorization. Postoperatively, her symptoms resolved completely, and follow-up imaging showed no recurrence. Tension pneumocephalus is a rare but serious complication of frontal sinus osteomas. Early diagnosis, combined with neuroimaging and surgical management, is essential for achieving favorable outcomes.

## Introduction

An osteoma is a benign (non-cancerous) bone tumor that most commonly arises from the skull or facial bones, particularly the paranasal sinuses (especially the frontal and ethmoid sinuses). Osteoma of the paranasal sinuses is a benign bony tumor, mainly involving the frontal and ethmoid sinuses. It is diagnosed most often in middle age (2nd to 5th decade) with a male preponderance, accounting for approximately 60-75% of cases [1].

Frontal sinus osteomas are benign, slow-growing tumors that are typically asymptomatic but may require surgical intervention depending on their size, location, or growth behavior. To aid clinical decision-making, several classification systems have been proposed in the literature. One commonly cited system classifies osteomas by size: small (<15 mm), medium (15-30 mm), and large (>30 mm). Larger lesions are more likely to cause symptoms or complications, such as orbital or intracranial extension. Another framework categorizes osteomas by location: anterior wall, posterior wall, or those involving the nasofrontal duct. This anatomical classification assists in anticipating potential complications and determining the most appropriate surgical approach, such as endoscopic resection for lesions near the duct versus open approaches for posteriorly located or extensive tumors. Additionally, osteomas may be characterized by their growth pattern, which can be exophytic, endophytic, or mixed, influencing both symptomatology and operative strategy. Including these classifications not only contextualizes the clinical significance of osteomas but also supports individualized treatment planning [2].

Pneumocephalus is the presence of air or gas within the intracranial cavity. It is usually associated with head injury and surgical interventions. Meningeal infections due to gas-forming organisms, barotrauma, otitis media, paranasal sinus tumors, and nasopharyngeal carcinoma are rare causes of pneumocephalus [2].

Pneumocephalus is an uncommon complication of long-standing osteomas; however, hemiparesis secondary to such pneumocephalus is a very rare complication [3]. In most cases, the intracranial air volume collected is less. However, if enough air accumulates within the intracranial cavity, tension pneumocephalus can develop, increasing intracranial pressure and causing neurological deficits, which necessitate emergency surgical intervention. In this report, we present a case of tension pneumocephalus due to frontal sinus osteomas.

## Case presentation

A 33-year-old female presented with a three-week history of progressive headache, followed by one week of right-sided hemiparesis affecting both the upper and lower limbs, graded as MRC grade 2 (active movement with gravity eliminated), corresponding to a modified Rankin scale score of 3. She denied any history of trauma, prior surgical intervention, or cerebrospinal fluid rhinorrhea. Neurological examination revealed right-sided weakness with no other focal deficits.

**Figure 1:**
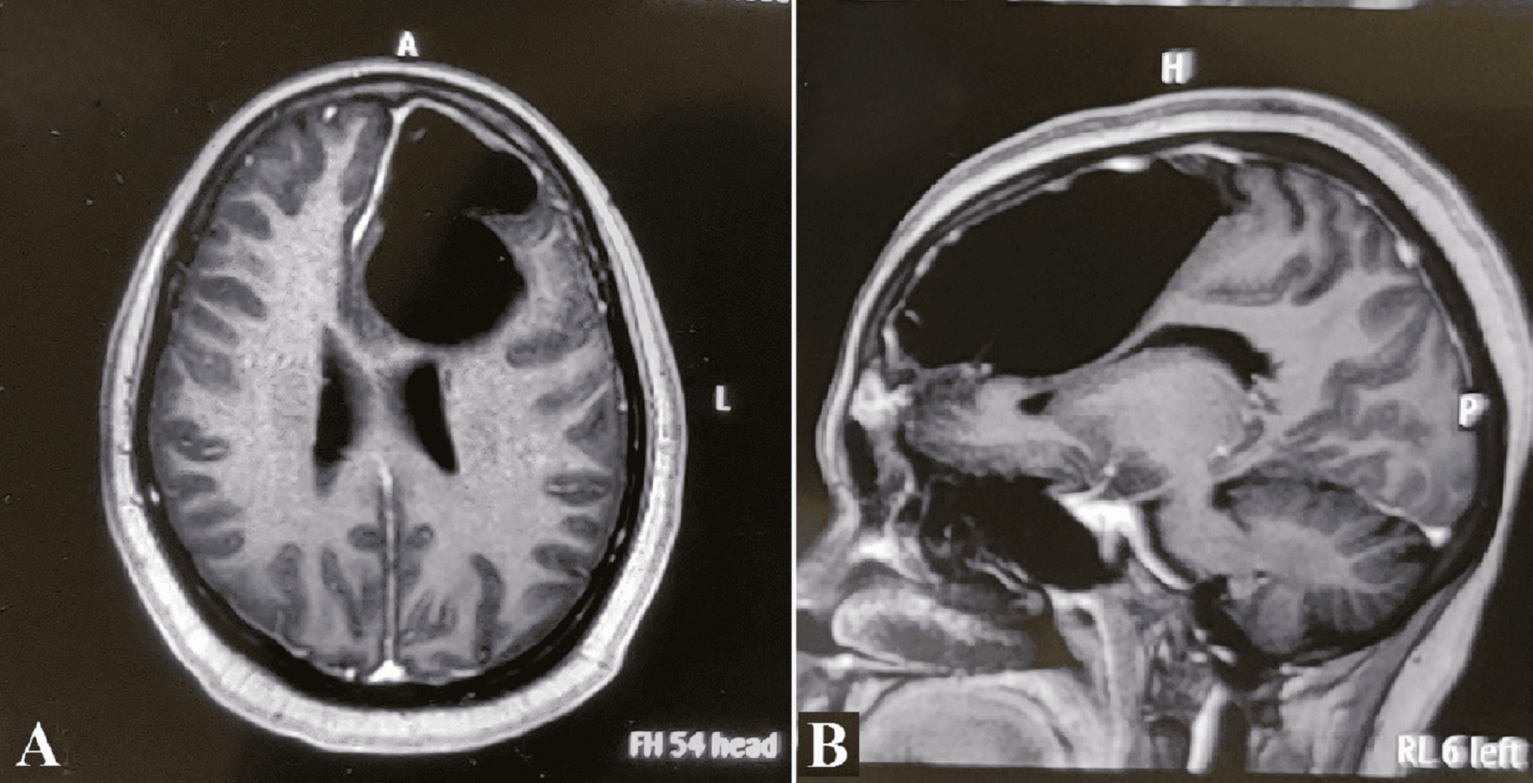
MRI brain T2-weighted axial image (A) and T1-weighted postcontrast sagittal image (B) reveal an isointense osseous lesion in the left frontal sinus, associated pneumocephalus without peripheral enhancement, and a mass effect on the left frontal lobe MRI: magnetic resonance imaging

MRI of the brain demonstrated a well-defined, intra-axial lesion in the left frontal lobe measuring 8.1 × 4.7 × 5.0 cm. The lesion appeared hypointense on T2-weighted and FLAIR images, non-enhancing on post-contrast T1-weighted sequences, and demonstrated blooming on gradient echo sequences, raising suspicion for a porencephalic cyst with hemorrhagic components. There was an associated mass effect on the ipsilateral lateral ventricle with effacement of adjacent sulci. Initial MRI revealed expansion of the left frontal sinus containing a non-enhancing lesion with intermediate signal intensity on both T1- and T2-weighted sequences, associated with scalloping of the outer cortex and a suspected defect in the inner table (Figure 1). These findings were initially interpreted as a possible porencephalic cyst with hemorrhagic components, leading to a differential diagnosis that included mucocele, fibrous dysplasia, or osteoma. However, subsequent CT imaging clarified the lesion’s dense osseous nature, and intraoperative findings confirmed the diagnosis of an osteoma. The initial MRI misinterpretation likely resulted from atypical imaging features and limited bone detail, which were later resolved through CT and surgical exploration.

**Figure 2:**
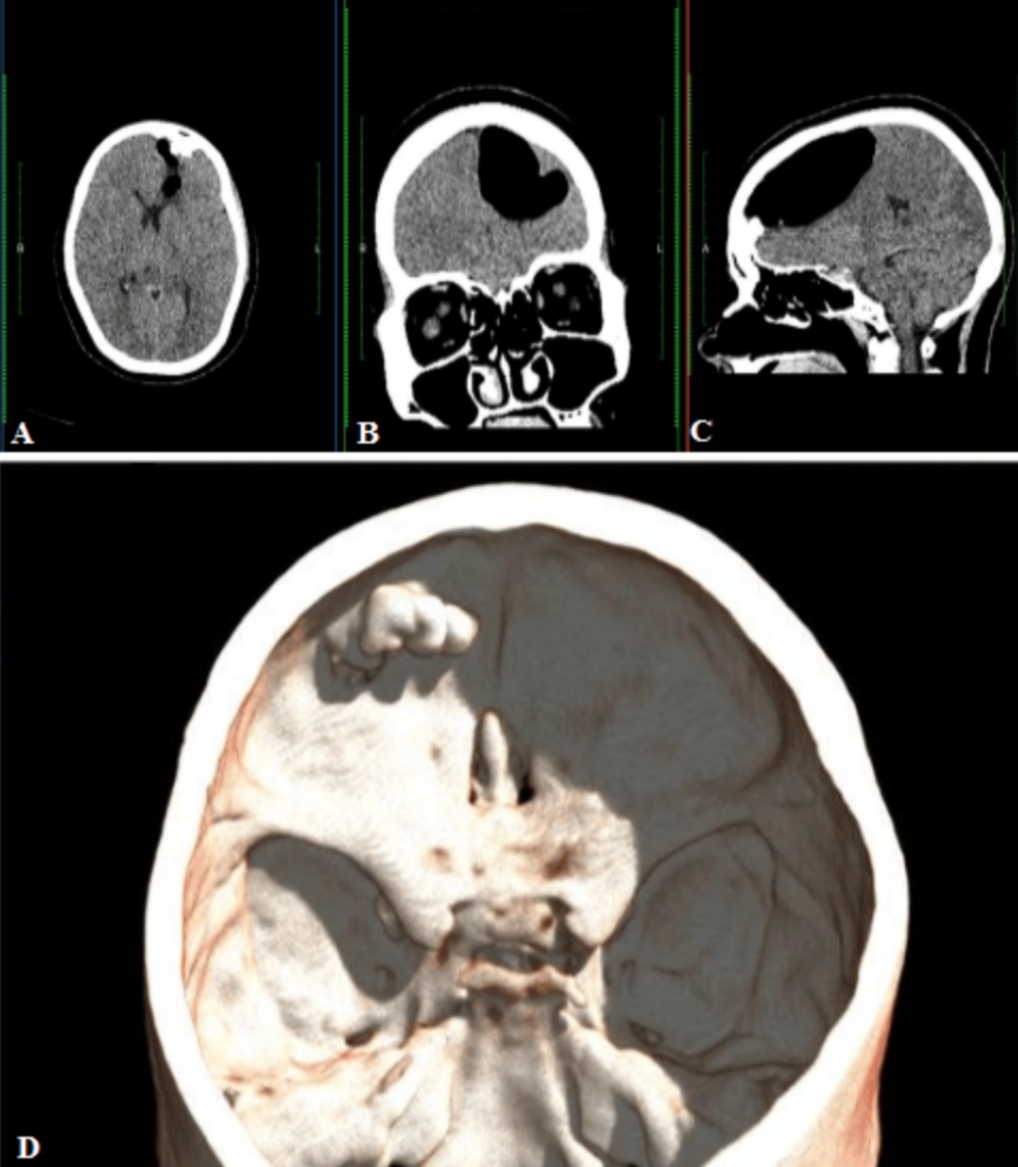
Non-enhanced CT brain axial (A), coronal (B), and sagittal (C) images showing a homogeneous bony mass within the left frontal sinus with associated pneumocephalus. 3D reconstruction image (D) showing the left frontal sinus osteoma from the superior aspect CT: computed tomography, 3D: three dimensional

Non-contrast CT of the head revealed a well-defined, lobulated, hyperdense bony lesion extending intracranially from the posterior wall of the left frontal sinus. A large intracranial air pocket was noted adjacent to the lesion in the left frontal region, suggestive of pneumocephalus (Figure 2).

**Figure 3:**
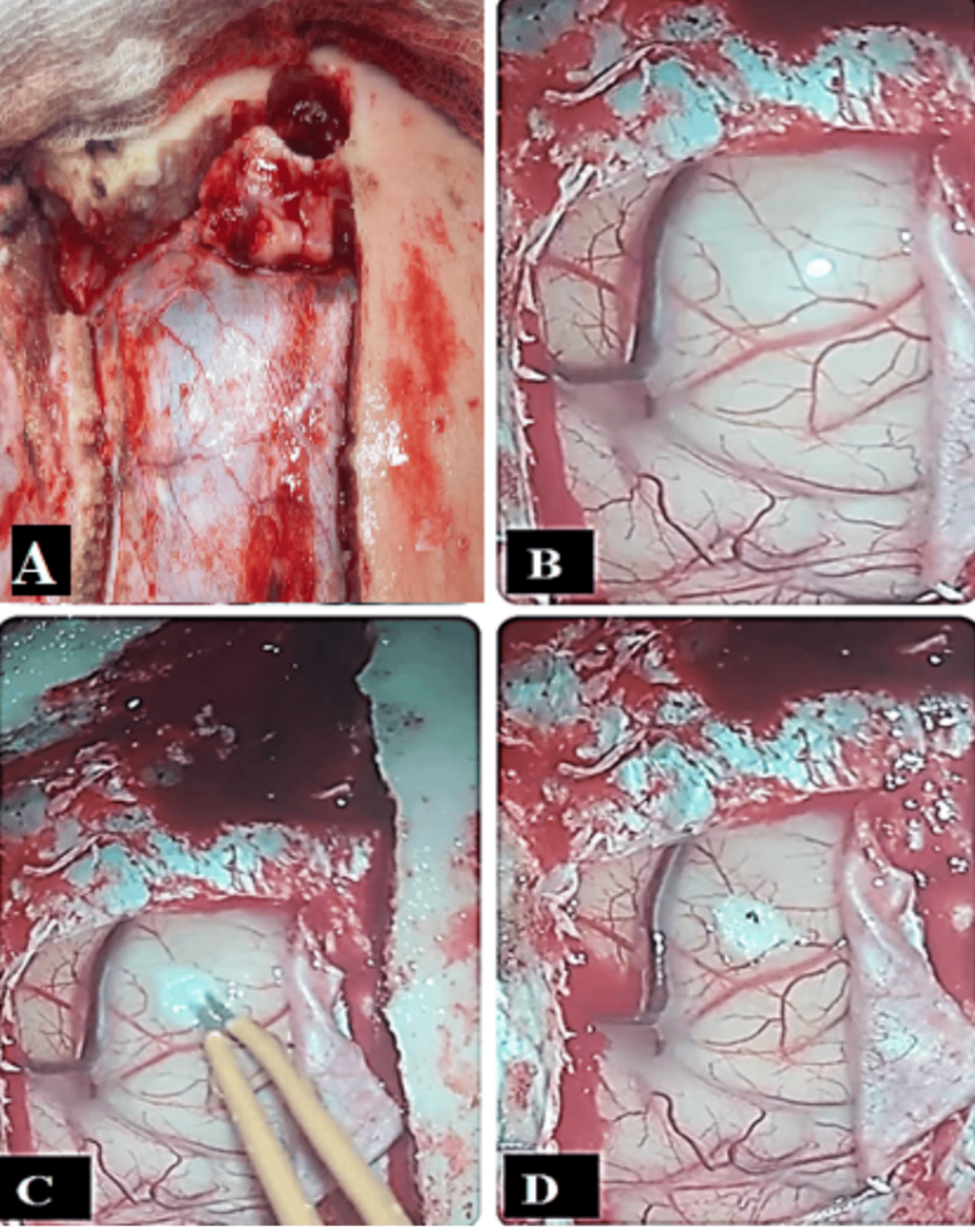
Intraoperative images showing the left frontal sinus osteoma (A); bulging of the left frontal lobe due to underlying pneumocephalus (B); small left frontal corticectomy performed using bipolar cautery (C); and deflation of the left frontal lobe pneumocephalus pocket with evacuation of air through the small corticectomy (D)

The patient underwent a left frontal craniotomy via a bicoronal incision. Intraoperatively, a bony tumor arising from the frontal sinus was found extending into the anterior cranial fossa and eroding the dura mater. The dura was tense and bulging. A small frontal corticectomy was performed to decompress the trapped air. The osteoma was excised completely, the dural defect was repaired, and the frontal sinus was exteriorized using a pedicled pericranial graft (Figure 3).

**Figure 4:**
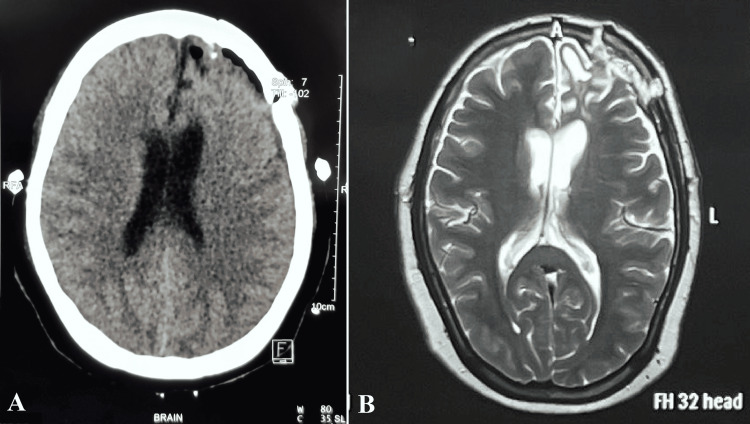
Postoperative axial (A) and sagittal (B) brain MRI images showing complete removal of the left frontal sinus osteoma and resolution of the pneumocephalus MRI: magnetic resonance imaging

**Figure 5:**
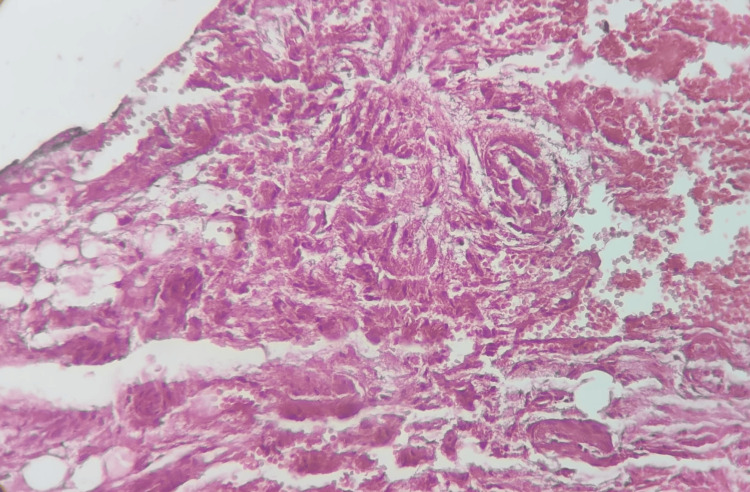
Hematoxylin and eosin staining of the osteoma nidus showing active osteoblasts surrounding the trabeculae of woven bone

Postoperatively, the patient experienced immediate improvement in hemiparesis, with complete neurological recovery achieved within five days. She remained free of complications and was discharged on postoperative day 7. Follow-up imaging confirmed total removal of the osteoma and complete resolution of the pneumocephalus (Figure 4). Histopathological examination confirmed the diagnosis of osteoma (Figure 5).

## Discussion

Frontal sinus osteomas are benign, slow-growing bone tumors commonly detected incidentally on imaging. While typically asymptomatic, complications may arise when these lesions erode the posterior table of the frontal sinus and breach the dura, allowing air to enter the intracranial space. This rare but potentially life-threatening situation can lead to pneumocephalus, or in severe cases, tension pneumocephalus, which manifests as neurological decline due to the mass effect from entrapped air.

A review of the literature reveals fewer than 10 well-documented cases (Table 1) implicating frontal or frontoethmoidal osteomas in the development of pneumocephalus [4-10]. Proposed pathophysiological mechanisms include the “ball-valve” effect, where a dural defect permits one-way ingress of air, and the establishment of communication between sinus mucosa and intracranial structures, sometimes triggered by air travel, barotrauma, or minor trauma [6,7].

**Table 1:** Published case reports of frontal osteoma with pneumocephalus ICP: intracranial pressure, CSF: cerebrospinal fluid, CT: computed tomography

Year	Study	Patient Presentation	Age/Gender	Findings	Outcome
2002	Johnson et al. [5]	Neurological symptoms due to intraparenchymal tension pneumatocele	62/male	Osteoma breached the dura; air entered the brain parenchyma	Surgical removal; full recovery
1989	Jackson et al. [6]	Non-specific headache and dizziness	51/male	Frontoethmoidal osteoma with pneumocephalus on CT	Excision; symptom resolution
2017	Hackenbroch et al. [7]	Headache, raised ICP symptoms	24/male	Ball-valve mechanism from frontal osteoma erosion	Endoscopic removal; favorable outcome
2017	Umredkar et al. [8]	Progressive hemiparesis	22/male	Extradural pneumocephalus due to posterior sinus wall erosion	Surgical decompression; neurological improvement
2019	Iplikcioglu et al. [9]	Tension pneumocephalus, altered mental status	24/male	Frontoethmoidal osteoma with dural defect	Endoscopic excision; no recurrence
2024	Candy et al. [10]	CSF leak, recurrent pneumocephalus	63/male, 27/male	Series of osteomas with minimally invasive management	Good recovery in all cases
2013	Lehmer et al. [11]	Chronic headache, tension pneumocephalus	30/male	Frontal osteoma with osteoblastoma-like histology	Surgical excision; histology confirmed variant
1998	Marras et al. [12]	Tension pneumocephalus	40/male	Frontal sinus osteoma with a fistulous connection between the sinus and the intracranial compartment	permanent frontal lobe damage, significant neurocognitive sequelae, seizures
2011	Guedes Bde et al. [13]	Progressive tension frontal headache lasting 20 days, gradual weakness of his right leg and arm, as well as tonic-clonic seizures	33/male	Large frontoethmoidal osteoma	Endoscopic excision; no recurrence

Clinical presentations range from nonspecific symptoms, such as headache, nausea, and dizziness, to more severe manifestations, including hemiparesis, in this case report attributed to the mass effect of tension pneumocephalus, as well as altered consciousness [7,8]. Diagnosis is most reliably made with computed tomography, which depicts intracranial air collections in extradural, subdural, or intraparenchymal compartments. MRI can provide complementary information, particularly regarding CSF leaks and soft tissue involvement.

Surgical management remains the cornerstone of treatment. Excision of the osteoma and meticulous dural repair are essential to preventing recurrence. Both open and endoscopic approaches have been reported in the literature, with endoscopic techniques favored in selected patients due to their minimally invasive nature and faster recovery [9]. In all reviewed cases, outcomes were favorable when complete resection and repair were achieved [5,8,9].

A noteworthy case described osteoblastoma-like histology within an osteoma, reinforcing the necessity of histopathological confirmation to rule out rare or aggressive variants [10]. Additionally, a recent case series from J Neurosurg Case Lessons (2024) emphasized the viability of endoscopic and minimally invasive approaches, showing successful outcomes when early diagnosis is achieved and appropriate surgical strategies are applied [9].

This case reinforces the importance of considering intracranial pneumocephalus in patients with known frontal sinus osteomas who present with new-onset neurological symptoms. Such patients, particularly those who become symptomatic after experiencing barometric changes or Valsalva maneuvers, should undergo prompt neuroimaging.

## Conclusions

Although osteomas of the paranasal sinuses are benign lesions, they may occasionally result in serious intracranial complications, including tension pneumocephalus. Timely neuroimaging follow-up is crucial in patients with known sinus osteomas, especially when neurological symptoms develop. When tension pneumocephalus is identified, prompt surgical intervention is crucial to prevent irreversible neurological damage and achieve full recovery.
